# Trends in online searching toward suicide pre-, during, and post the first wave of COVID-19 outbreak in China

**DOI:** 10.3389/fpsyt.2022.947765

**Published:** 2022-07-25

**Authors:** Hongguang Chen, Konglai Zhang, Hui Li, Mengqian Li, Shunfei Li

**Affiliations:** ^1^Peking University Sixth Hospital, Peking University Institute of Mental Health, NHC Key Laboratory of Mental Health (Peking University), National Clinical Research Center for Mental Disorders (Peking University Sixth Hospital), Beijing, China; ^2^Department of Epidemiology, Institute of Basic Medical Sciences, Chinese Academy of Medical Sciences, Beijing, China; ^3^Department of Psychosomatic Medicine, The First Affiliated Hospital of Nanchang University, Nanchang, China; ^4^Chinese PLA General Hospital, Beijing, China

**Keywords:** COVID-19, suicide, online searching behavior, depression, social psychiatry

## Abstract

COVID-19 may increase the risk of suicide, but the conclusion is still unclear. This study was designed to assess the impact of COVID-19 on suicide pre-, during, and post the first wave of COVID-19 in China. It was reported that online public searching was associated with their offline thoughts and behaviors. Therefore, this study was designed to explore the online search for suicide pre-, during, and post-COVID-19 in China. The keywords on suicide, COVID-19, unemployment, and depression were collected in 2019 and 2020 using the Baidu Search Index (BSI). A time-series analysis examined the dynamic correlations between BSI-COVID-19 and BSI-suicide. A generalized estimating equation model was used to calculate the coefficients of variables associated with the BSI-suicide. The BSI-suicide showed a significant increase (15.6%, *p* = 0.006) from the 5th to 9th week, which was also the point of the first wave of the COVID-19 outbreak. A time-series analysis between BSI-suicide and BSI-COVID-19 showed that the strongest correlation occurred at lag 1+ and lag 2+ week. In the pre-COVID-19 model, only BSI-depression was highly associated with BSI-suicide (β = 1.38, *p* = 0.008). During the COVID-19 model, BSI-depression (β = 1.77, *p* = 0.040) and BSI-COVID-19 (β = 0.03, *p* < 0.001) were significantly associated with BSI-suicide. In the post-COVID-19 model, BSI depression (β = 1.55, *p* = 0.010) was still highly associated with BSI-suicide. Meanwhile, BSI-unemployment (β = 1.67, *p* = 0.007) appeared to be linked to BSI-suicide for the first time. There was a surge in suicide-related online searching during the early stage of the first wave of the COVID-19 outbreak. Online suicide search volume peaked 1–2 weeks after the COVID-19 peak. The BSI of factors associated with suicide varied at different stages of the COVID-19 pandemic. The findings in this study are preliminary and further research is needed to arrive at evidence of causality.

## Introduction

COVID-19 has swept the world swiftly since being diagnosed and is still in its rage. COVID-19 has brought great disasters to humanity and would change the lifestyle of human beings in the short and long term, even if it can be controlled soon. Those changes will disturb our everyday life, work, and learning and pose tremendous pressure on all kinds of walks. Furthermore, it was reported that COVID-19 would significantly increase the risk of suicide in previous studies (1–3). Several factors underpinned these concerns, including a deterioration in population mental health, a higher prevalence of reported thoughts and behaviors of self-harm among people with COVID-19, problems accessing mental health services, and evidence suggesting that previous epidemics, such as severe acute respiratory syndrome (SARS), were associated with a rise in deaths by suicide (2, 4, 5). Widely reported studies modeling the effect of the covid-19 pandemic on suicide rates predicted increases ranging from 1 to 145%, mainly reflecting variation in underlying assumptions (6). Supposition is no replacement for evidence, and timely data on rates of suicide are vital. Several studies reporting suicide trends have emerged more recently. Overall, the literature on the effect of COVID-19 on suicide should be interpreted with caution (6). Nevertheless, a reasonably consistent picture is beginning to emerge from high-income countries. Some studies reported that the suicide rate did not increase at the early stages of the pandemic (7, 8) but most reported an increase in the suicide rate during the epidemic (2, 9, 10). The picture is much less evident in the middle- and low-income countries, where the safety nets available in better-resourced settings may be lacking. China should be the first country in the world to witness the entire process of pandemic control. Unfortunately, longitudinal epidemiological data on suicide have not been available in China. With the ubiquitous availability of the Internet, the collection and analysis of online searching behavior information have become a necessary supplement to predict the trends of “offline” behavior on suicide. Previous studies verified that *online* searching behaviors of the public were associated with their offline thoughts and behaviors (11–14). In this study, we tried to understand the suicide behaviors by analyzing their online search behaviors pre-, during, and post-COVID-19 pandemic in China.

## Materials and methods

The daily online search volume of suicide-related keywords during the first wave of COVID-19 from 1 January to 31 December 2020 was collected using the Baidu Search. As the most crucial searching tool in China, Baidu Search offers Baidu Index Service (BIS)^1^, as well as Google Trends, allowing the registered users to harness data on searched keywords. According to the 2021 Chinese search engine user behavior study report released by China Internet Network Information Center Reports, as of June 2021, China’s Internet users have reached *1.011 billion.* The number of search engine users has reached *795 million*, with the user penetration rate of Baidu search exceeding *90%*. The Baidu Search Index (BSI) was one of the functions of BIS, which was calculated by weighting the search frequency of each keyword in Baidu search according to the search volume of internet users and the keywords. The BSI of suicide-related terms was collected to comprehensively explore the factors associated with online searching behaviors, such as COVID-19, depression, and work status.

The detailed retrieval processes were as follows (keywords in Chinese, as shown in Supplementary Table 1): terms being used for searching would include: a combination of “COVID-19’ and “New Coronavirus” for COVID-19; “Suicide” and “Want to die” for suicide; “Depressed” for depression; “Unemployment” for working status. The search terms were matched by searching the keywords database of the Baidu Index Service according to searched contents. In addition, the selection of keywords referred to the demand graph analysis of searching keywords offered by the Baidu index. The demand graph analysis can show the demand for searched words shown by the changes in users’ searching behavior before and after the keyword search and can help in defining the exact keyword. The search content was determined with reference to the main socio-economic and psychosocial factors associated with suicide reported in previous studies. The retrieved areas covered the whole country. Furthermore, to further explore the impact of the COVID-19 pandemic on suicide-related keywords searching, the volumes of these keywords searched in the same period of 2019 were also collected. As a result, according to the epidemic of COVID-19 in China, the whole year of 2019 and the 1st–3rd week from the beginning of 2020 were defined as Pre-COVID-19; the timespan from lockdown to the reopening of Wuhan was defined as the time during the COVID-19 pandemic (from the 4th to 15th week of 2020); and the timespan from the reopening of Wuhan to the end of 2020 was defined as Post-COVID-19.

### Data analysis and statistics

The mean/median BSI was used to describe the average searching volume of those keywords for each week. A cross-correlogram for bivariate time series was employed further to demonstrate the dynamic correlations between BSI-COVID-19 and BSI-suicide contemporaneously and at various lagged values from the 1st to 53rd week in 2020. Granger Causality assumptions (15) have been verified and employed to verify the causal relationship between BSI-COVID-19 and BSI-suicide after the cross-correlation analysis. To identify changes in searching trends, joinpoint regression was estimated for every keyword using the Joinpoint Regression Program, Version 4.9.0.0. The Joinpoint Regression Program is a trend analysis software developed by the U.S. National Cancer Institute to analyze data from the Surveillance Epidemiology and End Results Program (16). The joinpoint regression model is designed to describe continuous changes and uses the grid-search method to fit the regression function with unknown joinpoints assuming constant variance and uncorrelated errors. The number of significant joinpoints was identified by performing several permutation tests, each of which has a correct significance level asymptotically. Each *p*-value is found using Monte Carlo methods, and the overall asymptotic significance level is maintained through a Bonferroni correction. The Joinpoint Regression Program performs a series of hypothesis tests that test the null hypothesis of k_a_ joinpoints against the alternative hypothesis of k_b_ joinpoints, where k_a_ and k_b_ change for each hypothesis test. Each *p*-value corresponds to this type of test. The *p*-value is an estimate of the probability, under the assumption that there are only k_a_ joinpoints, of observing data that look more like a joinpoint model with greater than k_a_ joinpoints than the data we have observed. This method describes changes in data trends by connecting several line segments on a log scale at “joinpoints.”

In addition, a weekly percent change (WPC) in BSIs for each line segment and the corresponding 95% confidence interval (*CI*) were estimated. Finally, the WPC is tested to determine whether a difference exists from the null hypothesis of no change (0%). In the final model, each joinpoint informs a statistically significant change in trends (increase or decrease), and each of those trends is described by a WPC. The Generalized Estimating Equation (GEE) model was used to estimate the coefficient (95% *CI*) of variables associated with the BSI-suicide between 2019 and 2020. The database was constructed with EpiData v. 3.1 (EpiData Association, Denmark), and data were analyzed using SPSS v. (SPSS, Inc., Chicago, IL, United States).

## Results

### Baidu Search Index for suicide between 2019 and 2020

**FIGURE 1:**
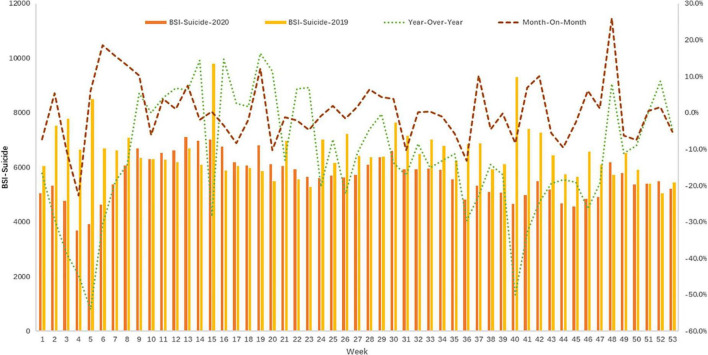
Time trends for the weekly Baidu Search Index (BSI)-suicide in 2019 and 2020.

Considering the development and control of the COVID-19 pandemic in China, this study described and compared BSIs weekly. There were 53 weeks, and BSI for suicide ranged from 3,692 to 7,109 in 2020 and 5,051 to 9,788 in 2019. Compared with the same period in 2019, the BSI-suicide increased sharply from the 5th to 9th week in 2020, with the year-on-year growth rate soaring from −54 to 5.4%. It fluctuated from the 10th week and decreased gradually in the following weeks. However, it began to pick up slightly from the 46th week to the end of the year (as shown in Figure 1). The month-over-month fluctuations in 2020 were also up and down, especially at the year’s beginning and end.

### Trends in Baidu Search Index-suicide and related factors between 2019 and 2020 according to the joinpoints identified by the analysis

Table 1 presents the WPC of BSI-suicide and related factors in China between 2019 and 2020. The BSI-suicide significantly decreased from the 1st to 5th week (−7.5%, *p* = 0.016), increased from the 5th to 9th week (15.6%, *p* = *0.006*), and dropped from the 9th to 45th week (−0.9%, *p* < *0.001*) in 2020. However, the whole year of 2019 showed a slow downward trend (−0.2%, *p* = *0.044*). The BSI-depression significantly decreased from the 1st to 4th week (−8.3%, *p* = *0.015*), increased from the 4th to 11th week (8.8%, *p* < *0.001*), and slightly decreased from the 11th to 25th week (−0.9%, *p* = *0.012*) and from the 28th to 51st week (−2.26%, *p* < *0.001*) in 2020. The trend for the BSI-depression in 2019 showed a fluctuating state with an extensive range. Both BSIs for unemployment significantly decreased from the 1st to 4th week, both in 2019 (−15.3%, *p* = 0.023) and 2020 (−19.7%, *p* = 0.002) but increased significantly from the 4th to 15th week (16.0%, *p* < 0.001) and slightly decreased from the 15th to 30th week (−5.3%, *p* < 0.001) in 2020. Consistent with the development of COVID-19, the BSI-COVID-19 increased significantly from the 1st to 6th week (1,243.4%, *p* < 0.001), followed by a slightly decreased trend from the 6th to 53rd week (−1.5%, *p* = 0.05).

**TABLE 1:** Trends in Baidu Search Index (BSI)-suicide and associated factors from 1st to 53rd week both in 2019 and 2020^†^.

	Segments	2019				2020			
		Week	WPC^a^	95%CI		Week	WPC	95%CI	
				Lower	Upper			Lower	Upper
Suicide	Trend 1	1–53	−0.2*	−0.5	−0.0	1–5	−7.5*	−13.2	−1.5
	Trend 2	–	–	–	–	5–9	15.6**	4.6	27.7
	Trend 3	–	–	–	–	9–45	−0.9***	−1.2	−0.7
	Trend 4	–	–	–	–	45–53	1.6	−0.6	3.9
Depression	Trend 1	1–17	3.0***	2.2	3.9	1–4	−8.3*	−14.4	−1.8
	Trend 2	17–30	−2.6***	−3.8	−1.5	4–11	8.8***	6.3	11.3
	Trend 3	30–33	13.2	−7.9	39.0	11–25	−0.9*	−1.7	−0.2
	Trend 4	33–38	−5.7	−11.7	0.6	25–28	6.5	−7.1	22.3
	Trend 5	38–41	18.7	−3.4	45.8	28–51	−2.3***	−2.6	−1.9
	Trend 6	41–53	−5.0***	−6.2	−3.8	51–53	8.6	−5.4	24.6
COVID-19	Trend 1	–	–	–	–	1–6	1,243.4***	751.8	2,018.8
	Trend 2	–	–	–	–	6–53	−1.5*	−3.1	0.0
Unemployment	Trend 1	1–4	−15.3*	−26.5	−2.4	1–4	−19.7**	−29.9	−8.0
	Trend 2	4–7	17.4	−11.6	55.8	4–15	16.0***	13.6	18.5
	Trend 3	7–53	−0.4**	−0.6	−0.1	15–30	−5.3***	−6.5	−4.1
	Trend 4	–	–	–	–	30–53	0.2	−0.4	0.9

^a^WPC, weekly percentage change; 95%CI, 95% confidence interval; *P < 0.05, **P < 0.01, ***P < 0.001. ^†^Segment data can meet the assumptions of the log-linear model.

### Cross-correlation and causal relationship between Baidu Search Index-suicide and Baidu Search Index-COVID-19

**FIGURE 2:**
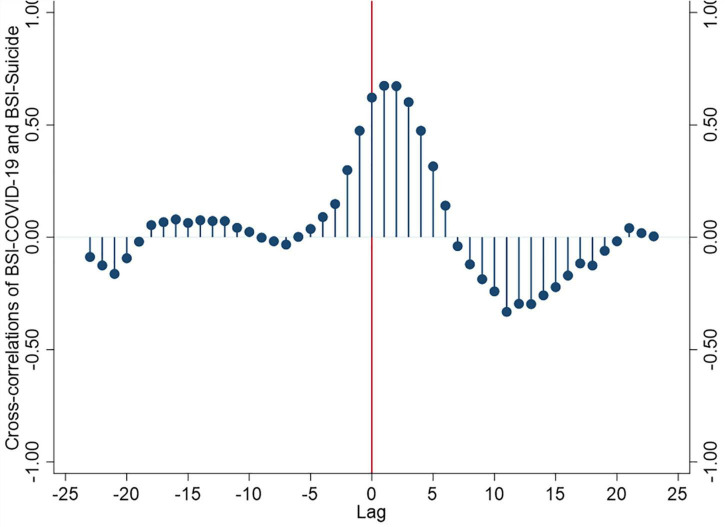
A cross-correlation function for the weekly BSI-COVID-19 and BSI-suicide in 2020.

A time-series analysis on the dynamic correlations between BSI-suicide and BSI-COVID-19 showed that the strongest correlation occurred at lag 1+ and 2+ (as shown in Figure 2 and Supplementary Figure 1), suggesting that the peak BSI-COVID-19 can lead to peak BSI-suicide 1 or 2 weeks later. In addition, the cross-correlations from lags +3 to lags +7, lags −1 to −4, and lags −11 to −18 showed different degrees of the positive correlation between BSI-COVID-19 and BSI-suicide. However, the cross-correlations from lags +7 to +19 and lags −20 to lags −23 suggested that the BSI-COVID-19 and BSI-suicide were negatively related over time. Varsoc was used to estimate the optional lags and found that all likelihood, final prediction error (FPE), Akaike information criterion (AIK), Hannan–Quinn information criterion (HQIC), and Schwarz–Bayesian information criterion (SBIC) had chosen a model with one lag. Granger causality Wald tests showed that the lagged values of BSI-COVID19 caused BSI-suicide (χ^2^ = 8.192, *p* = 0.004) and lagged values of BSI-suicide did not cause BSI-COVID-19 (χ^2^ = 8.192, *p* = 0.004). The direction of causality is from BSI-COVID-19 to BSI-suicide.

### Factors associated with Baidu Search Index-suicide pre-, during, and post COVID-19

To explore potential factors associated with BSI-suicide, overall and subgroup GEE analyses stratified by the control of the COVID-19 pandemic were performed. The findings of the overall GEE analysis showed that BSI-depression was associated with BSI-suicide (regression coefficient, β = 0.99, *p* = 0.001, Table 2) under the condition that BSI-suicide significantly decreased over time (regression coefficient, β = −13.87, *p* = 0.003). In the pre-COVID-19 model, only BSI-depression was positively associated with BSI-suicide over time (regression coefficient, β = 1.38, *p* = 0.008). During the COVID-19 model, BSI-depression (regression coefficient, β = 1.77, *p* = 0.040) and BSI-COVID-19 (regression coefficient, β = 0.03, *p* < 0.001) showed a highly significant association with BSI-suicide. In the post-COVID-19 model, BSI-depression (regression coefficient, β = 1.55,p *P* = 0.010) was still highly associated with BSI-suicide. Meanwhile, BSI-unemployment (regression coefficient, β = 1.67, *p* = 0.007) was linked to BSI-suicide for the first time.

**TABLE 2:** Associations between Baidu Search Index (BSI)-COVID-19 and BSI-suicide pre-, during, and post-COVID-19 pandemic in China*.

Variables	Overall		Stratified by COVID-19 epidemic					
			Pre-COVID-19		During COVID-19 pandemic		Post-COVID-19	
	β (95%CI)	*P*	β (95%CI)	*P*	β (95%CI)	*P*	β (95%CI)	*P*
BSI-depression	0.99 (0.40–1.58)	0.001	1.38 (0.36–2.41)	0.008	1.77 (0.08–3.46)	0.040	1.55 (0.37–2.72)	0.010
BSI-unemployment	0.96 (−0.07 to 2.00)	0.067	−1.43 (−6.40 to 3.54)	0.574	0.49 (−0.78 to 1.76)	0.446	1.67 (0.46–2.88)	0.007
Time (week)	−13.87 (−23.00 to −4.74)	0.003	−16.42 (−33.54 to 0.70)	0.060	27.03 (−197.59 to 251.64)	0.814	4.88 (−25.77 to 35.53)	0.755
BSI-COVID-19	0.01 (0.00–0.03)	0.097	–	–	0.03 (0.02–0.04)	<0.001	−0.01 (−0.03 to 0.01)	0.259

*GEE model (family = gaussian; link = identity; corr = exchangeable).

## Discussion

This study was the first to explore the impact of the first wave of the COVID-19 outbreak on suicide through longitudinal online searching data in China. Previous studies had reported that online public searching could be a potential predictor for their offline thoughts and behaviors (11, 13). With the deep integration of Internet use behavior into daily life, people tend to retrieve information and help from the Internet, especially in the face of sudden public health time, such as COVID-19. Therefore, network behavior analysis can be used as a supplementary monitoring means of suicidal behavior during the epidemic.

The study found a surge in BSI-suicide during the early COVID-19 outbreak and the peak searching volume for COVID-19, especially from the 5th to 9th week. Since the 10the week, BSI-suicide began to show a downward trend, also a turning point for China’s COVID-19 control. A national online cross-sectional survey performed between 28 February and 11 March 2020, reported that the prevalence of suicidal ideation in China during the COVID-19 outbreak was as high as 16.4% (17). Another online survey involving 1,172 participants found that the high risk of suicide and behavior prevalence was 2.8% (18). A meta-analysis study suggested that increased event rates for suicide ideation were 10.81%, suicide attempts 4.68%, and self-harm 9.63%, respectively, during the COVID-19 pandemic (2). An exciting finding was the lag effect that the peak of BSI-suicide was 1–2 weeks later than the peak of BSI-COVID-19. The above discovery might be related to understanding the COVID-19 pandemic for the public. In the early stage of the COVID-19 pandemic, more attention was paid to the disease’s infectivity and severity. After a preliminary understanding of the risk, the search for emotional problems, such as panic, stress, and depression, would occur in a short period, which would lead to an increase in the search for suicide. This process took about 1–2 weeks, indicating the critical time window for suicide prevention. Previous studies have reported that mental health consequences of the COVID-19 crisis, including suicidal behavior, are likely to peak later than the actual pandemic (3, 19).

Consistent with the fact that depression makes substantial contributions to suicide across the lifespan (20–22), online search behavior obtained the same conclusion that BSI-depression was strongly associated with BSI-suicide at any stage of the COVID-19 epidemic. This finding also confirmed that the online search behavior could effectively reflect netizens’ offline thoughts and behaviors.

In addition, the regression coefficient also indicated that the association between BSI-depression and BSI-suicide was strengthened during the COVID-19 pandemic, suggesting that coping with depression problems should be sustained and strengthened. Furthermore, the subgroup analysis showed that BSI-COVID-19 contributed to BSI-suicide only during the COVID-19 outbreak period, which might be related to the effective control of the pandemic in China soon after the outbreak. Finally, it is worth noting that BSI unemployment was associated with BSI-suicide and BSI-depression in the post-COVID-19 period. As for the relationship between depression and suicide, previous studies have reported the association between unemployment and suicide during or not during the COVID-19 pandemic (7, 17, 22, 23). These findings suggest that in the post-COVID-19 era, the focus of suicide prevention needs to be expanded or adjusted from COVID-19-induced mental problems to a comprehensive scope, such as unemployment in this study (23). Those changes should be recognized and valued. Notably, in this study, the correlations between keyword searching behavior to suicide and other related factors were consistent with the results of population questionnaires in previous studies (3, 24). It also confirms the possibility of mutual predictability between online retrieval behavior and offline behavior. Previous studies have reported that mass media have played a critical role in spreading the COVID-19 and addressing netizens’ online searching interest (25–27), thus favoring the increasing searching for COVID-19. Meanwhile, it can also spread panic and fear among the public, increasing the online searching for psychological symptoms, such as suicide-related words. Moreover, searching for depression and unemployment was also affected by mass media. Therefore, it is noted that the online search relationship between suicide-related keywords and other keywords might be strengthened by related mass media report. This study, based on a 2-year longitudinal keyword online searching of more than *700 million* netizens, explored the impact of COVID-19 on suicide and factors associated with BSI-suicide over time. However, this study had several limitations. First, this study only analyzed the effects of COVID-19 on suicide through online searching behaviors, which could not replace the online or offline questionnaire investigation. Still, it can provide valuable references and supplement for understanding of the impact of COVID-19 on suicide. In addition, participants only included netizens, but not those who never surf the Internet or those who surf the Internet but without searching behaviors, even though they were in the minority. Second, due to the availability of the search keywords in the database, only a few representative factors related to suicide were analyzed. Third, factors associated with suicide search behaviors might differ across countries and over time, depending on conditions, such as the control of the epidemic, the capacity of existing mental health services, and suicide prevention programs, making the extrapolation of the results cautious. Finally, the seasonal factor might affect the online search amount during the study period.

## Conclusion

Under the condition that the COVID-19 pandemic was brought under control quickly, peak BSI-suicide occurred 1–2 weeks later than the pandemic outbreak and gradually decreased over time. BSI-depression had always contributed to BSI-suicide regardless of COVID-19, but this contribution had increased during the outbreak phase. In the post-COVID-19 era, further attention should be paid to other factors associated with BSI-suicide, such as BSI-unemployment, which might be related to the later effect of the epidemic on the economy. In addition, online searching of keywords related to suicide varied at different stages of the COVID-19 pandemic. The findings in this study are preliminary and further research is needed to arrive at evidence of causality. Nevertheless, these findings provide another profile of suicide among netizens pre-, during, and post-COVID-19 epidemic and underscore the significance of policy-making and suicide prevention in public health emergencies. These findings suggest that in the post-COVID-19 era, the focus of suicide prevention needs to be expanded or adjusted from COVID-19-induced mental problems to a comprehensive scope, such as unemployment in this study. Those changes should be recognized and valued.

## Data availability statement

The raw data supporting the conclusions of this article will be made available upon reasonable request to the corresponding author.

## Author contributions

HC and SL: concept and design, had full access to all the data in the study, takes responsibility for the integrity of the data, and the accuracy of the data analysis. HC and HL: acquisition, analysis, or interpretation of data. HC and KZ: drafting of the manuscript. HC: statistical analysis. SL: obtained funding. KZ and ML: administrative, technical, or material support. ML and HC: supervision. All authors critically revised the manuscript for important intellectual content, contributed to the article, and approved the submitted version.

## Footnotes

1
https://index.baidu.com/v2/index.html#/
