# Supplementary figures and images for: Trends in online searching toward suicide pre-, during, and post the first wave of COVID-19 outbreak in China
Source: Front Psychiatry. 2022 Jul 25;13:947765. doi: 10.3389/fpsyt.2022.947765 (PMC9357924; doi:10.3389/fpsyt.2022.947765)

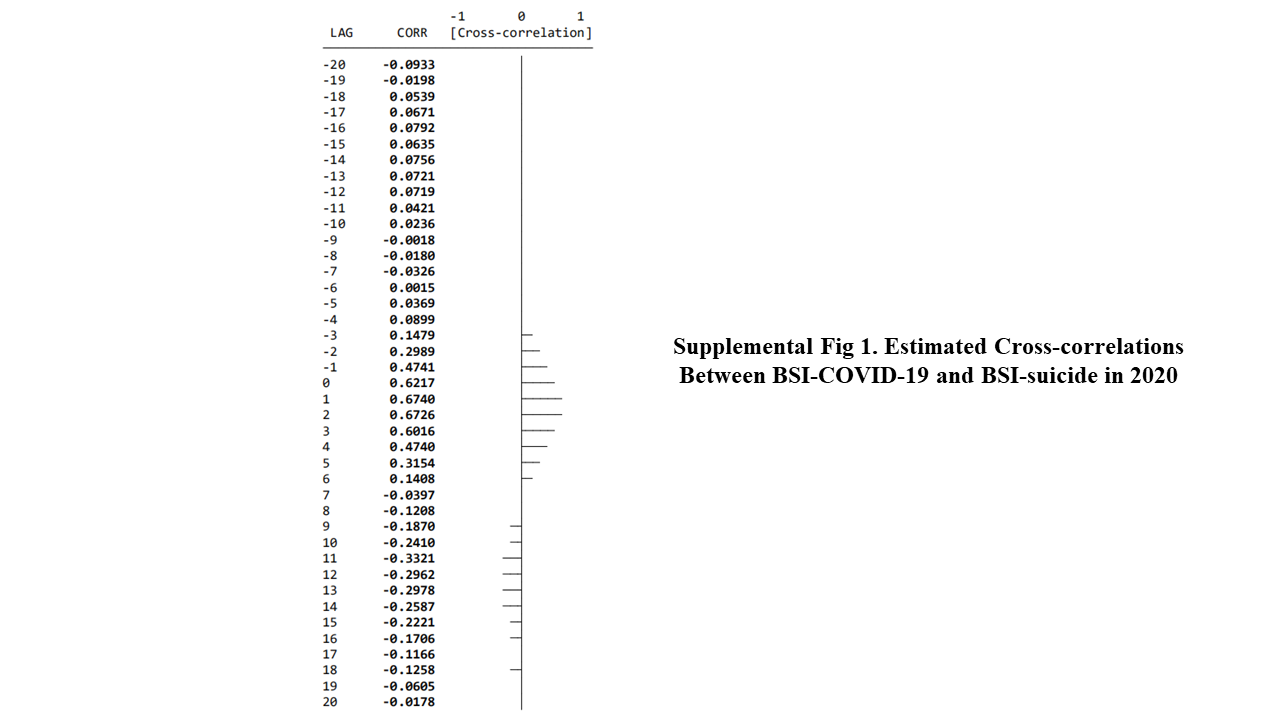

Supplement: Supplementary file 1 [file Image_1.TIF]
