# Supplementary material for: Trends in online searching toward suicide pre-, during, and post the first wave of COVID-19 outbreak in China
Source: Front Psychiatry. 2022 Jul 25;13:947765. doi: 10.3389/fpsyt.2022.947765 (PMC9357924; doi:10.3389/fpsyt.2022.947765)
Supplement: Supplementary file 2 [file Table_1.DOCX]

**Supplemental Table 1 Searching keywords in Chinese**

| **Searching contents** | **Keywords (In Chinese)** |
| --- | --- |
| Suicide | “自杀”+“想死” |
| Depression | “抑郁” |
| Unemployment | “失业” |
| COVID-19 | “新冠肺炎”+“COVID-19” |
